# Supplementary material for: Mobile phone use intention while driving among public service vehicle drivers: Magnitude and its social and cognitive determinants
Source: PLoS One. 2021 Apr 30;16(4):e0251007. doi: 10.1371/journal.pone.0251007 (PMC8087078; doi:10.1371/journal.pone.0251007)
Supplement: Attachment — Submitted filename: Response to Reviewers.docx [file pone.0251007.s002.docx]

To: PLOS ONE academic editor and reviewers

Thank you for reviewing my article entitled “Mobile phone use intention while driving among public service vehicle drivers: Magnitude and its social and cognitive determinants.” The points mentioned by the academic editor and reviewers are invaluable. So, I have revised the article by considering the comments.

I. **Response to academic editor’s comment**

**Comments:**

Journal Requirements: When submitting your revision, we need you to address these additional requirements.

1. Please ensure that your manuscript meets PLOS ONE's style requirements, including those for file naming by attaching the addresses of PLOS ONE style templates.
2. We note that you have indicated that data from this study are available upon request. PLOS only allows data to be available upon request if there are legal or ethical restrictions on sharing data publicly. In your revised cover letter, please address the following prompts:
   a. If there are ethical or legal restrictions on sharing a de-identified data set, please explain them in detail (e.g., data contain potentially sensitive information, data are owned by a third-party organization, etc.) and who has imposed them (e.g., an ethics committee). Please also provide contact information for a data access committee, ethics committee, or other institutional body to which data requests may be sent.
   b. If there are no restrictions, please upload the minimal anonymized data set necessary to replicate your study findings as either Supporting Information files or to a stable, public repository and provide us with the relevant URLs, DOIs, or accession numbers.
3. Please amend either the title on the online submission form (via Edit Submission) or the title in the manuscript so that they are identical.

**Responses:**

Thank you so much for your invaluable comments. Responses for each comment are outlined here under.

1. Regarding journal requirements, I have tried to address the requirements based on the PLOS ONE style sample templates.
2. Concerning data availability statement, I previously stated that the data from this study are available upon reasonable request with the rationale that I did not receive consent from study participants to share the data set for web. Unless this ethical issue restricts me, I can upload the dataset to replicate the findings in supporting information files. The data is owned by me.
3. As far as title difference in the online submission and the manuscript, I have amended the title on the online submission form.
4. I have also included ethical approval and consent to participate in the methods section. Thank you again.
II. **Responses to reviewer #1:**

**Comments:**

 This paper investigates the mobile phone use while driving for drivers of public service vehicles in Ethiopia. The research topic is interesting and worth of investigation. The manuscript is well organized. Nonetheless, I would like to see the formulation of the proposed hierarchical regression model and illustrate how the hierarchical structure is considered. Moreover, a comparison between the hierarchical regression model and other alternatives is suggested to demonstrate the strength of the proposed model. Besides, it would be expected to point out the practical implications of the findings.

**Responses:**

I would like to thank the reviewer for such comments. As per the comments, I have proposed and illustrated the hierarchical regression model. Besides, standard multiple regression, hierarchical regression and stepwise regression models were compared so as to show the strength of the hierarchical regression model. Finally, in the conclusions section, I have tried to point out the practical implications of the findings.

III. **Responses to reviewer #2:**

Comments:

The current paper aims at examining the contributing factors towards mobile phone use while driving using questionnaire study. There are several issues with the paper:
1. The significance of the study should be deleted. The stated significance of the study is a bit of a stretch and does not reflect the unique contribution of the current paper.
2. The literature review is not exhaustive, the following paper is suggested to be cited and discussed in the paper:
[1] Feng Chen, Mingtao Song and Xiaoxiang Ma, Investigation on the Injury Severity of Drivers in Rear-End Collisions Between Cars Using a Random Parameters Bivariate Ordered Probit Model, International Journal of Environmental Research and Public Health, 2019, 16(14) , 2632.
[2] Shao Xiaojun, Ma Xiaoxiang, Chen Feng, Song Mingtao, Pan Xiaodong, You Kesi, 2020. A random parameters ordered probit analysis of injury severity in truck involved rear-end collisions. International journal of environmental research and public health. doi:10.3390/ijerph17020395

**Responses:**

I also would like to thank the reviewer for the helpful comments. In line with the comments, I have deleted the significance of the study by pointing out its practical implications of the findings in the conclusion section. I have also discussed the empirical findings from suggested recent articles in relation to my study.
