# Supplementary material for: Mobile phone use intention while driving among public service vehicle drivers: Magnitude and its social and cognitive determinants
Source: PLoS One. 2021 Apr 30;16(4):e0251007. doi: 10.1371/journal.pone.0251007 (PMC8087078; doi:10.1371/journal.pone.0251007)
Supplement: Attachment — Submitted filename: Response to Reviewers2.docx [file pone.0251007.s003.docx]

To: PLOS ONE academic editor and reviewers

Thank you so much for reviewing my article entitled “Mobile phone use intention while driving among public service vehicle drivers: Magnitude and its social and cognitive determinants.” The points mentioned by the academic editor and reviewers are invaluable. So, I have revised the article by considering the comments.

I. **Response to academic editor’s comment**

**Comments:**

Journal Requirements: Please review your reference list to ensure that it is complete and correct. If you have cited papers that have been retracted, please include the rationale for doing so in the manuscript text, or remove these references and replace them with relevant current references. Any changes to the reference list should be mentioned in the rebuttal letter that accompanies your revised manuscript. If you need to cite a retracted article, indicate the article’s retracted status in the References list and also include a citation and full reference for the retraction notice.

**Responses:**

Thank you so much for your helpful comments. As per your comments, I have reviewed my reference list and found one omitted reference, but available in the in text citation. I have added the omitted source in the reference section, namely. East Gojjam Zone Police (2013) Traffic Accidents Statistics in East Gojjam Zone. I have also included two sources in data analysis section and in the reference list as well based upon the reviewer’s comment.

Thank you again.

II. **Responses to reviewer #1:**

**Comments:**

Reviewer #1: Most of my comments have been addressed properly. A minor suggestion is that more references on the Pearson correlation test and hierarchical model should be cited in the Data analysis section.
**Responses:**

I would like to appreciate the reviewer for the invaluable comments. As per your comments, I have discussed the empirical findings from suggested recent articles in relation to my study.
